# Trimethyl­sulfonium 1-amino-6-fluoro-2,3,4,5,7,8,9,10,11,12-decaiodo-1-carba-*closo*-dodeca­borate

**DOI:** 10.1107/S1600536812004424

**Published:** 2012-02-10

**Authors:** Maik Finze, Guido J. Reiss

**Affiliations:** a Institut für Anorganische Chemie, Julius-Maximilians-Universität Würzburg, Am Hubland, D-97074 Würzburg, Germany; b Institut für Anorganische Chemie und Strukturchemie, Lehrstuhl II: Material- und Strukturforschung, Heinrich-Heine-Universität Düsseldorf, Universitätsstrasse 1, D-40225 Düsseldorf, Germany

## Abstract

In the asymmetric unit of the title salt, C_3_H_9_S^+^·CH_2_B_11_FI_10_N^−^ or (CH_3_)_3_S[1-H_2_N-6-F-*closo*-1-CB_11_I_10_], both ions lie in general positions. The anion is perfectly ordered and so the positions of the C—NH_2_ vertex and the fluorine substituent are clearly assigned. The relatively short C—N bond length may be inter­preted in terms of a very electron deficient {*closo*-1-CB_11_} cluster.

## Related literature

For a general overview on monocarba-*closo*-dodeca­borates, see: Körbe *et al.* (2006 ▶). For the synthesis and properties of 1-amino­monocarba-*closo*-dodeca­boron clusters, see: Jelínek *et al.* (1986 ▶); Srivastava *et al.* (1996 ▶); Finze (2007 ▶, 2009 ▶); Finze *et al.* (2007 ▶); Finze & Sprenger (2010 ▶). For studies on the proton affinity of halogenated {*closo*-1-CB_11_} clusters, see: Himmelspach *et al.* (2011 ▶, 2012 ▶). For the formation of (CH_3_)_3_S^+^ from dimethyl sulfoxide, see: Nifontova & Lavrentiev (1993 ▶); Forrester *et al.* (1995 ▶); Park *et al.* (2005 ▶). For the structure of (CH_3_)_3_SBr, see: Svensson & Kloo (1996 ▶).
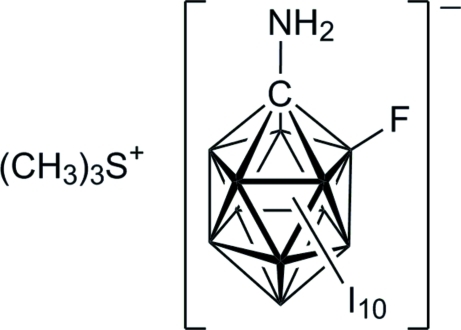


## Experimental

### 

#### Crystal data

C_3_H_9_S^+^·CH_2_B_11_FI_10_N^−^
*M* *_r_* = 1512.11
Monoclinic, 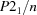

*a* = 10.0672 (1) Å
*b* = 16.7057 (2) Å
*c* = 17.5574 (2) Å
β = 93.175 (1)°
*V* = 2948.26 (6) Å^3^
*Z* = 4
Mo *K*α radiation
μ = 10.59 mm^−1^
*T* = 100 K
0.79 × 0.28 × 0.20 mm

#### Data collection

Oxford Diffraction Xcalibur Eos diffractometer
Absorption correction: analytical [*CrysAlis PRO* (Oxford Diffraction, 2009 ▶), based on expressions derived by Clark & Reid (1995 ▶)] *T*_min_ = 0.040, *T*_max_ = 0.204
30584 measured reflections
5482 independent reflections
5282 reflections with *I* > 2σ(*I*)
*R*_int_ = 0.029

#### Refinement

*R*[*F* ^2^ > 2σ(*F* ^2^)] = 0.024
*wR*(*F* ^2^) = 0.052
*S* = 1.31
5482 reflections
264 parameters
2 restraints
H atoms treated by a mixture of independent and constrained refinement
Δρ_max_ = 1.06 e Å^−3^
Δρ_min_ = −0.61 e Å^−3^

### 

Data collection: *CrysAlis PRO* (Oxford Diffraction, 2009 ▶); cell refinement: *CrysAlis PRO*; data reduction: *CrysAlis PRO*; program(s) used to solve structure: *SHELXS97* (Sheldrick, 2008 ▶); program(s) used to refine structure: *SHELXL97* (Sheldrick, 2008 ▶); molecular graphics: *DIAMOND* (Brandenburg, 2011 ▶); software used to prepare material for publication: *publCIF* (Westrip, 2010 ▶).

## Supplementary Material

Crystal structure: contains datablock(s) I, global. DOI: 10.1107/S1600536812004424/ru2027sup1.cif

Structure factors: contains datablock(s) I. DOI: 10.1107/S1600536812004424/ru2027Isup2.hkl

Additional supplementary materials:  crystallographic information; 3D view; checkCIF report

## Figures and Tables

**Fig. 1.**
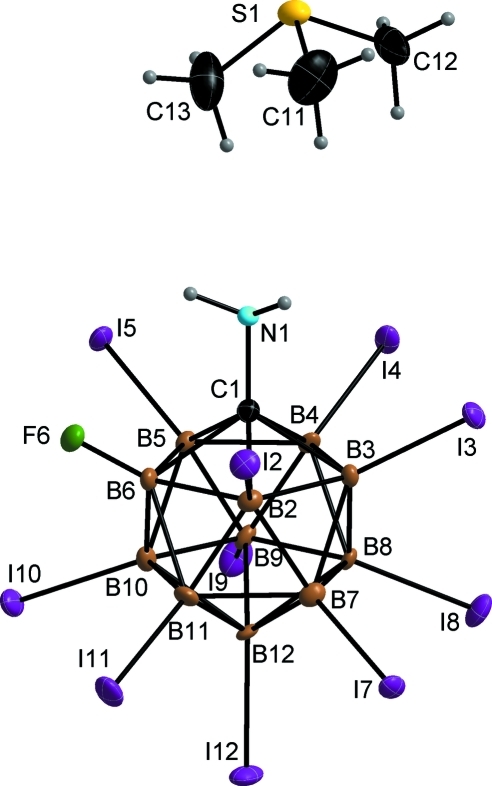
The structure of the asymmetric unit, showing 50% probability displacement ellipsoids. Hydrogen atoms are shown as spheres of arbitrary radius.

## supplementary crystallographic information

### Comment

Monocarba-*closo*-dodecaborates with amino groups that are bonded to the cluster carbon or boron atoms are potential building blocks for a broad range of applications (Körbe *et al.*, 2006). The properties of the amino group are strongly influenced by (i) the other substituents of the {*closo*-1-CB_11_} cluster (Jelínek *et al.* 1986; Srivastava *et al.* 1996; Finze *et al.*, 2007; Finze, 2007) and (ii) the type of cluster atom that it is bonded to (Finze, 2009).

Halogenation of all boron vertices of the {*closo*-1-CB_11_} cluster (Körbe *et al.*, 2006) results in a decrease of the electron density in the cluster. For example, this effect is evident from the calculated proton affinity of [*closo*-1-CB_11_*X*_11_]^2–^ that strongly decreases from *X* = H to *X* = halogen (Himmelspach *et al.*, 2012). A further example is the coordination of CH_3_CN or H_2_O to Hg^II^ of the dianionic mercury(II) complex [Hg(*closo*-1-CB_11_F_11_)_2_]^2–^, which is related to the Lewis-acidity of mercury (Himmelspach *et al.*, 2011). In contrast, for the complex [Hg(*closo*-1-CB_11_H_11_)_2_]^2–^ that possess a lower Lewis-acidity at the mercury atom no coordination of a third ligand was observed (Himmelspach *et al.*, 2012). Similarly, a much lower electron density at the amino group is found for halogenated {1-H_2_N-*closo*-1-CB_11_} clusters in comparison to [1-H_2_N-*closo*-1-CB_11_H_11_]^-^: The non-halogenated anion is easily protonated to yield 1-H_3_N-*closo*-1-CB_11_H_11_ (p*K*_a_ = 6.0) (Jelínek *et al.* 1986; Finze, 2009) while the attempted protonation of [1-H_2_N-6-F-*closo*-1-CB_11_I_10_]^-^ with conc. hydrochloric acid failed (Finze & Sprenger, 2010).

Treatment of a solution of H(solv)[1-H_2_N-6-F-*closo*-1-CB_11_I_10_] in dimethyl sulfoxide (DMSO) with methanol and conc. hydrochloric acid results in the slow formation of crystals of the trimethylsulfonium salt Me_3_S[1-H_2_N-6-F-*closo*-1-CB_11_I_10_]. Similar reactions of DMSO to result in trimethylsulfonium salts were reported earlier (*e.g.* Nifontova & Lavrentiev, 1993; Forrester *et al.*, 1995; Park *et al.*, 2005).

The title compound trimethylsulfonium-1-amino-6-fluoro-1-carba-*closo*-dodecaborate (Figure 1) crystallizes in the monoclinic space group *P*2_1_/*n* with one formula unit in the asymmetric unit. The position of the C–NH_2_ and the B–F vertex of the [1-H_2_N-6-F-*closo*-1-CB_11_I_10_]^-^ anion are clearly assigned (i) from comparative refinements and (ii) the C–N and B–F as well as the inner-cluster C–B bond lengths are similar to values reported for [1-H_2_N-*closo*-1-CB_11_F_11_]^-^ and [1-H_2_N-6-HO-*closo*-1-CB_11_F_10_]^-^ (Finze *et al.*, 2007). The bond lengths and angles of the trimethylsulfonium cation are similar to those reported for other (CH_3_)_3_S^+^ salts, for example (CH_3_)_3_SBr (Svensson & Kloo, 1996).

### Experimental

[Et_4_N][1-H_2_N-6-F-*closo*-1-CB_11_I_10_] (50 mg, 0.03 mmol), which was synthesized according to a published procedure (Finze & Sprenger, 2010), was suspended in a mixture of aqueous HCl (50 ml, 10% *v*/*v*) and diethyl ether (50 ml). After 30 minutes of stirring the solid dissolved. The ethereal layer was separated and the aqueous solution was extracted with Et_2_O (2 *x* 20 ml). The combined ether phases were dried with MgSO_4_, filtered, and most of the diethyl ether was removed under reduced pressure. DMSO was added (2 ml) and the residual Et_2_O was removed under reduced pressure. Methanol (2 ml) and conc. hydrochloric acid (4 ml) was added. According to ^11^B{^1^H}-NMR spectroscopy the [1-H_2_N-6-F-*closo*-1-CB_11_I_10_]^-^ anion was not protonated. After 3 month crystals of the title compound were obtained from the reaction mixture. ^1^H NMR (500.13 MHz, CD_2_Cl_2_): *d* = 2.90 p.p.m. (s, (CH_3_)_3_S^+^) (the signal of the protons of the amino group was not observed as a result of signal broadening). ^11^B NMR (160.46 MHz, CD_2_Cl_2_): *d* = -1.6 (s, 1 B, BF, B-2), -14.3 (s, 1 B, B-12), -17.1 (s, 2 B, B-4 and B-5), -18.9 (s, 2 B, B-8 and B-10), -18.9 (s, 2 B, B-3 and B-6), -19.2 (s, 2 B, B-7 and B-11), -21.0 (s, 1 B, B-9). IR (ATR): n_as_(NH_2_) 3362 *versus*, n_s_(NH_2_) 3297 cm^-1^*versus*.

### Refinement

All hydrogen atoms of the CH_3_ groups were refined using a riding model with the *U*_iso_(H) set to 1.5*U*_eq_(C). The coordinates of the two hydrogen atoms at the nitrogen atom were refined unrestrictedly together with one refined common U_iso_ value. Anisotropic displacement parameters of all non-hydrogen atoms were also refined unrestrictedly.

### Crystal data

**Table:**

C_3_H_9_S^+^·CH_2_B_11_FI_10_N^−^	*F*(000) = 2608
*M**_r_* = 1512.11	*D*_x_ = 3.407 Mg m^−^^3^
Monoclinic, *P*2_1_/*n*	Mo *K*α radiation, λ = 0.71073 Å
Hall symbol: -P 2yn	Cell parameters from 35017 reflections
*a* = 10.0672 (1) Å	θ = 3.0–34.0°
*b* = 16.7057 (2) Å	µ = 10.59 mm^−^^1^
*c* = 17.5574 (2) Å	*T* = 100 K
β = 93.175 (1)°	Block, colourless
*V* = 2948.26 (6) Å^3^	0.79 × 0.28 × 0.20 mm
*Z* = 4	

### Data collection

**Table:**

Oxford Diffraction Xcalibur Eos diffractometer	5482 independent reflections
Radiation source: fine-focus sealed tube	5282 reflections with *I* > 2σ(*I*)
Equatorial mounted graphite monochromator	*R*_int_ = 0.029
Detector resolution: 16.2711 pixels mm^-1^	θ_max_ = 25.5°, θ_min_ = 3.2°
ω scans	*h* = −12→12
Absorption correction: analytical [*CrysAlis PRO* (Oxford Diffraction, 2009), based on expressions derived by Clark & Reid (1995)]	*k* = −20→20
*T*_min_ = 0.040, *T*_max_ = 0.204	*l* = −21→21
30584 measured reflections	

### Refinement

**Table:**

Refinement on *F*^2^	Secondary atom site location: difference Fourier map
Least-squares matrix: full	Hydrogen site location: inferred from neighbouring sites
*R*[*F*^2^ > 2σ(*F*^2^)] = 0.024	H atoms treated by a mixture of independent and constrained refinement
*wR*(*F*^2^) = 0.052	*w* = 1/[σ^2^(*F*_o_^2^) + (0.014*P*)^2^ + 16.*P*] where *P* = (*F*_o_^2^ + 2*F*_c_^2^)/3
*S* = 1.31	(Δ/σ)_max_ = 0.001
5482 reflections	Δρ_max_ = 1.06 e Å^−^^3^
264 parameters	Δρ_min_ = −0.61 e Å^−^^3^
2 restraints	Extinction correction: *SHELXL97* (Sheldrick, 2008), Fc^*^=kFc[1+0.001xFc^2^λ^3^/sin(2θ)]^-1/4^
Primary atom site location: structure-invariant direct methods	Extinction coefficient: 0.000548 (19)

### Special details

**Table:**

Experimental. CrysAlisPro, Oxford Diffraction (2009). Analytical numeric absorption correction using a multifaceted crystal model based on expressions derived by Clark & Reid (1995).
Geometry. All e.s.d.'s (except the e.s.d. in the dihedral angle between two l.s. planes) are estimated using the full covariance matrix. The cell e.s.d.'s are taken into account individually in the estimation of e.s.d.'s in distances, angles and torsion angles; correlations between e.s.d.'s in cell parameters are only used when they are defined by crystal symmetry. An approximate (isotropic) treatment of cell e.s.d.'s is used for estimating e.s.d.'s involving l.s. planes.
Refinement. Refinement of *F*^2^ against ALL reflections. The weighted *R*-factor *wR* and goodness of fit *S* are based on *F*^2^, conventional *R*-factors *R* are based on *F*, with *F* set to zero for negative *F*^2^. The threshold expression of *F*^2^ > σ(*F*^2^) is used only for calculating *R*-factors(gt) *etc*. and is not relevant to the choice of reflections for refinement. *R*-factors based on *F*^2^ are statistically about twice as large as those based on *F*, and *R*- factors based on ALL data will be even larger.

### Fractional atomic coordinates and isotropic or equivalent isotropic displacement parameters (Å^2^)

**Table:**

	*x*	*y*	*z*	*U*_iso_*/*U*_eq_	
C1	0.2112 (5)	0.3730 (3)	0.5075 (3)	0.0111 (10)	
N1	0.2183 (4)	0.4462 (3)	0.4682 (2)	0.0107 (9)	
H12	0.292 (4)	0.475 (4)	0.480 (4)	0.034 (14)*	
H11	0.148 (5)	0.478 (4)	0.475 (4)	0.034 (14)*	
B2	0.2708 (6)	0.3639 (4)	0.6030 (3)	0.0105 (11)	
I2	0.34637 (3)	0.46793 (2)	0.661845 (19)	0.01491 (9)	
B3	0.3549 (6)	0.3180 (4)	0.5266 (3)	0.0087 (11)	
I3	0.54383 (3)	0.36148 (2)	0.494809 (19)	0.01425 (9)	
B4	0.2327 (6)	0.2837 (4)	0.4561 (3)	0.0090 (11)	
I4	0.27883 (3)	0.28803 (2)	0.338027 (18)	0.01482 (9)	
B5	0.0752 (6)	0.3128 (3)	0.4868 (3)	0.0088 (11)	
I5	−0.08352 (3)	0.35589 (2)	0.411424 (19)	0.01391 (8)	
B6	0.0985 (6)	0.3608 (4)	0.5781 (3)	0.0094 (11)	
F6	0.0191 (3)	0.42395 (19)	0.59388 (17)	0.0160 (7)	
B7	0.3288 (6)	0.2642 (4)	0.6136 (3)	0.0112 (12)	
I7	0.49137 (3)	0.23918 (2)	0.694333 (18)	0.01296 (8)	
B8	0.3054 (6)	0.2145 (4)	0.5231 (3)	0.0092 (11)	
I8	0.44339 (4)	0.12326 (2)	0.49050 (2)	0.01707 (9)	
B9	0.1296 (6)	0.2120 (4)	0.4979 (3)	0.0105 (11)	
I9	0.03159 (4)	0.11836 (2)	0.43173 (2)	0.02016 (9)	
B10	0.0465 (6)	0.2599 (4)	0.5729 (3)	0.0105 (11)	
I10	−0.15349 (3)	0.23210 (2)	0.60119 (2)	0.01709 (9)	
B11	0.1677 (6)	0.2908 (4)	0.6452 (3)	0.0104 (11)	
I11	0.12763 (4)	0.29664 (2)	0.763929 (19)	0.01921 (9)	
B12	0.1890 (6)	0.1985 (3)	0.5955 (3)	0.0089 (11)	
I12	0.17344 (4)	0.08517 (2)	0.65238 (2)	0.01909 (9)	
S1	0.16683 (16)	0.54368 (9)	0.20914 (9)	0.0228 (3)	
C11	0.2577 (8)	0.5579 (5)	0.2974 (5)	0.044 (2)	
H111	0.2125	0.5962	0.3275	0.066*	
H112	0.3452	0.5773	0.2883	0.066*	
H113	0.2647	0.5079	0.3243	0.066*	
C12	0.2548 (8)	0.4642 (4)	0.1672 (4)	0.0377 (18)	
H121	0.3398	0.4833	0.1528	0.057*	
H122	0.2047	0.4450	0.1228	0.057*	
H123	0.2675	0.4214	0.2034	0.057*	
C13	0.0275 (7)	0.4896 (4)	0.2388 (5)	0.0403 (19)	
H131	0.0574	0.4445	0.2689	0.060*	
H132	−0.0246	0.4712	0.1948	0.060*	
H133	−0.0258	0.5238	0.2687	0.060*	

### Atomic displacement parameters (Å^2^)

**Table:**

	*U*^11^	*U*^22^	*U*^33^	*U*^12^	*U*^13^	*U*^23^
C1	0.011 (3)	0.011 (3)	0.011 (2)	0.001 (2)	0.001 (2)	0.000 (2)
N1	0.009 (2)	0.010 (2)	0.013 (2)	0.0001 (17)	0.0016 (17)	0.0037 (18)
B2	0.012 (3)	0.009 (3)	0.011 (3)	−0.001 (2)	−0.001 (2)	0.000 (2)
I2	0.01739 (18)	0.01209 (18)	0.01497 (17)	−0.00177 (13)	−0.00148 (13)	−0.00448 (13)
B3	0.007 (3)	0.011 (3)	0.008 (3)	0.001 (2)	0.001 (2)	−0.001 (2)
I3	0.00892 (16)	0.01894 (19)	0.01509 (17)	−0.00289 (13)	0.00248 (13)	0.00286 (14)
B4	0.010 (3)	0.012 (3)	0.005 (2)	0.003 (2)	−0.002 (2)	0.000 (2)
I4	0.01385 (17)	0.0234 (2)	0.00719 (16)	0.00384 (14)	0.00041 (12)	−0.00055 (13)
B5	0.008 (3)	0.008 (3)	0.011 (3)	0.001 (2)	−0.001 (2)	0.000 (2)
I5	0.01020 (17)	0.01890 (19)	0.01234 (16)	0.00449 (13)	−0.00204 (13)	0.00323 (13)
B6	0.006 (3)	0.012 (3)	0.011 (3)	0.001 (2)	0.001 (2)	0.001 (2)
F6	0.0159 (16)	0.0151 (16)	0.0170 (16)	0.0052 (13)	0.0020 (12)	−0.0007 (13)
B7	0.012 (3)	0.010 (3)	0.012 (3)	0.000 (2)	−0.003 (2)	−0.001 (2)
I7	0.01068 (17)	0.01716 (19)	0.01059 (16)	0.00013 (13)	−0.00340 (12)	0.00326 (13)
B8	0.007 (3)	0.010 (3)	0.011 (3)	0.004 (2)	0.000 (2)	0.001 (2)
I8	0.01617 (18)	0.01587 (19)	0.01883 (18)	0.00825 (14)	−0.00215 (14)	−0.00469 (14)
B9	0.008 (3)	0.010 (3)	0.013 (3)	0.003 (2)	−0.003 (2)	−0.001 (2)
I9	0.01760 (19)	0.01479 (19)	0.0273 (2)	−0.00160 (14)	−0.00620 (15)	−0.00826 (15)
B10	0.007 (3)	0.015 (3)	0.010 (3)	0.000 (2)	0.000 (2)	0.000 (2)
I10	0.00822 (17)	0.0216 (2)	0.02161 (18)	−0.00206 (14)	0.00219 (13)	0.00644 (15)
B11	0.013 (3)	0.013 (3)	0.006 (3)	−0.001 (2)	0.002 (2)	0.002 (2)
I11	0.01868 (19)	0.0299 (2)	0.00953 (16)	0.00059 (15)	0.00519 (13)	0.00235 (15)
B12	0.008 (3)	0.006 (3)	0.012 (3)	0.002 (2)	−0.001 (2)	0.004 (2)
I12	0.01749 (18)	0.01344 (19)	0.0260 (2)	−0.00212 (14)	−0.00231 (15)	0.01093 (15)
S1	0.0301 (8)	0.0173 (7)	0.0215 (7)	0.0052 (6)	0.0055 (6)	0.0044 (6)
C11	0.034 (4)	0.044 (5)	0.052 (5)	−0.004 (3)	−0.017 (4)	−0.013 (4)
C12	0.051 (5)	0.022 (4)	0.044 (4)	0.011 (3)	0.032 (4)	0.003 (3)
C13	0.024 (4)	0.030 (4)	0.067 (5)	−0.007 (3)	0.011 (3)	−0.020 (4)

### Geometric parameters (Å, º)

**Table:**

C1—N1	1.409 (7)	B7—B8	1.796 (8)
C1—B5	1.721 (8)	B7—B12	1.799 (8)
C1—B3	1.730 (7)	B7—B11	1.798 (8)
C1—B6	1.738 (7)	B7—I7	2.146 (6)
C1—B2	1.756 (8)	B8—B12	1.796 (8)
C1—B4	1.764 (8)	B8—B9	1.800 (8)
N1—H12	0.898 (10)	B8—I8	2.161 (6)
N1—H11	0.896 (10)	B9—B10	1.788 (8)
B2—B6	1.767 (8)	B9—B12	1.797 (8)
B2—B7	1.772 (8)	B9—I9	2.155 (6)
B2—B11	1.788 (8)	B10—B11	1.787 (8)
B2—B3	1.797 (8)	B10—B12	1.791 (8)
B2—I2	2.140 (6)	B10—I10	2.151 (6)
B3—B4	1.791 (8)	B11—B12	1.791 (8)
B3—B8	1.799 (8)	B11—I11	2.148 (6)
B3—B7	1.804 (8)	B12—I12	2.150 (6)
B3—I3	2.139 (6)	S1—C13	1.770 (7)
B4—B9	1.771 (8)	S1—C11	1.771 (7)
B4—B5	1.771 (8)	S1—C12	1.778 (6)
B4—B8	1.778 (8)	C11—H111	0.9600
B4—I4	2.150 (5)	C11—H112	0.9600
B5—B9	1.778 (8)	C11—H113	0.9600
B5—B10	1.787 (8)	C12—H121	0.9600
B5—B6	1.795 (8)	C12—H122	0.9600
B5—I5	2.143 (6)	C12—H123	0.9600
B6—F6	1.361 (7)	C13—H131	0.9600
B6—B10	1.765 (8)	C13—H132	0.9600
B6—B11	1.774 (8)	C13—H133	0.9600
			
N1—C1—B5	117.7 (4)	B11—B7—B3	108.3 (4)
N1—C1—B3	119.5 (4)	B2—B7—I7	119.1 (4)
B5—C1—B3	112.1 (4)	B8—B7—I7	123.5 (4)
N1—C1—B6	120.2 (4)	B12—B7—I7	124.0 (4)
B5—C1—B6	62.5 (3)	B11—B7—I7	120.8 (3)
B3—C1—B6	111.8 (4)	B3—B7—I7	121.0 (4)
N1—C1—B2	121.3 (4)	B4—B8—B12	107.7 (4)
B5—C1—B2	112.1 (4)	B4—B8—B7	108.3 (4)
B3—C1—B2	62.1 (3)	B12—B8—B7	60.1 (3)
B6—C1—B2	60.7 (3)	B4—B8—B3	60.1 (3)
N1—C1—B4	118.3 (4)	B12—B8—B3	108.0 (4)
B5—C1—B4	61.1 (3)	B7—B8—B3	60.2 (3)
B3—C1—B4	61.6 (3)	B4—B8—B9	59.3 (3)
B6—C1—B4	111.7 (4)	B12—B8—B9	59.9 (3)
B2—C1—B4	111.8 (4)	B7—B8—B9	107.9 (4)
C1—N1—H12	114 (5)	B3—B8—B9	107.3 (4)
C1—N1—H11	113 (5)	B4—B8—I8	122.0 (3)
H12—N1—H11	108 (7)	B12—B8—I8	122.5 (3)
C1—B2—B6	59.1 (3)	B7—B8—I8	120.3 (3)
C1—B2—B7	105.9 (4)	B3—B8—I8	120.4 (3)
B6—B2—B7	108.2 (4)	B9—B8—I8	123.8 (4)
C1—B2—B11	106.2 (4)	B4—B9—B5	59.9 (3)
B6—B2—B11	59.9 (3)	B4—B9—B10	108.2 (4)
B7—B2—B11	60.7 (3)	B5—B9—B10	60.2 (3)
C1—B2—B3	58.3 (3)	B4—B9—B12	108.0 (4)
B6—B2—B3	107.4 (4)	B5—B9—B12	107.9 (4)
B7—B2—B3	60.7 (3)	B10—B9—B12	59.9 (3)
B11—B2—B3	109.0 (4)	B4—B9—B8	59.7 (3)
C1—B2—I2	119.0 (3)	B5—B9—B8	107.3 (4)
B6—B2—I2	117.5 (4)	B10—B9—B8	107.6 (4)
B7—B2—I2	127.4 (4)	B12—B9—B8	59.9 (3)
B11—B2—I2	123.6 (4)	B4—B9—I9	121.9 (4)
B3—B2—I2	122.5 (4)	B5—B9—I9	119.9 (3)
C1—B3—B4	60.1 (3)	B10—B9—I9	120.2 (4)
C1—B3—B2	59.7 (3)	B12—B9—I9	122.8 (4)
B4—B3—B2	108.6 (4)	B8—B9—I9	124.3 (4)
C1—B3—B8	106.1 (4)	B6—B10—B11	59.9 (3)
B4—B3—B8	59.4 (3)	B6—B10—B5	60.7 (3)
B2—B3—B8	107.1 (4)	B11—B10—B5	108.7 (4)
C1—B3—B7	105.6 (4)	B6—B10—B9	108.4 (4)
B4—B3—B7	107.4 (4)	B11—B10—B9	108.8 (4)
B2—B3—B7	58.9 (3)	B5—B10—B9	59.6 (3)
B8—B3—B7	59.8 (3)	B6—B10—B12	107.7 (4)
C1—B3—I3	121.0 (4)	B11—B10—B12	60.1 (3)
B4—B3—I3	121.0 (3)	B5—B10—B12	107.8 (4)
B2—B3—I3	120.8 (3)	B9—B10—B12	60.3 (3)
B8—B3—I3	124.5 (3)	B6—B10—I10	118.2 (3)
B7—B3—I3	124.3 (3)	B11—B10—I10	120.6 (3)
C1—B4—B9	105.6 (4)	B5—B10—I10	120.0 (3)
C1—B4—B5	58.3 (3)	B9—B10—I10	123.8 (4)
B9—B4—B5	60.3 (3)	B12—B10—I10	125.0 (4)
C1—B4—B8	105.6 (4)	B6—B11—B10	59.4 (3)
B9—B4—B8	61.0 (3)	B6—B11—B12	107.3 (4)
B5—B4—B8	108.6 (4)	B10—B11—B12	60.1 (3)
C1—B4—B3	58.3 (3)	B6—B11—B2	59.5 (3)
B9—B4—B3	109.0 (4)	B10—B11—B2	107.0 (4)
B5—B4—B3	107.1 (4)	B12—B11—B2	107.4 (4)
B8—B4—B3	60.6 (3)	B6—B11—B7	106.7 (4)
C1—B4—I4	120.3 (3)	B10—B11—B7	107.6 (4)
B9—B4—I4	125.9 (4)	B12—B11—B7	60.2 (3)
B5—B4—I4	122.0 (3)	B2—B11—B7	59.2 (3)
B8—B4—I4	123.9 (3)	B6—B11—I11	121.8 (4)
B3—B4—I4	118.6 (3)	B10—B11—I11	123.0 (3)
C1—B5—B4	60.6 (3)	B12—B11—I11	123.1 (4)
C1—B5—B9	107.1 (4)	B2—B11—I11	121.0 (4)
B4—B5—B9	59.9 (3)	B7—B11—I11	122.1 (3)
C1—B5—B10	105.9 (4)	B11—B12—B10	59.9 (3)
B4—B5—B10	108.2 (4)	B11—B12—B8	108.5 (4)
B9—B5—B10	60.2 (3)	B10—B12—B8	107.7 (4)
C1—B5—B6	59.2 (3)	B11—B12—B9	108.3 (4)
B4—B5—B6	108.7 (4)	B10—B12—B9	59.8 (3)
B9—B5—B6	107.5 (4)	B8—B12—B9	60.2 (3)
B10—B5—B6	59.0 (3)	B11—B12—B7	60.1 (3)
C1—B5—I5	119.4 (3)	B10—B12—B7	107.4 (4)
B4—B5—I5	123.6 (3)	B8—B12—B7	60.0 (3)
B9—B5—I5	126.9 (4)	B9—B12—B7	107.9 (4)
B10—B5—I5	122.6 (3)	B11—B12—I12	121.2 (3)
B6—B5—I5	117.2 (3)	B10—B12—I12	122.0 (3)
F6—B6—C1	117.9 (4)	B8—B12—I12	121.8 (3)
F6—B6—B10	125.1 (4)	B9—B12—I12	121.6 (4)
C1—B6—B10	106.2 (4)	B7—B12—I12	122.1 (3)
F6—B6—B2	120.4 (5)	C13—S1—C11	101.0 (4)
C1—B6—B2	60.1 (3)	C13—S1—C12	99.3 (3)
B10—B6—B2	109.0 (4)	C11—S1—C12	102.5 (4)
F6—B6—B11	126.1 (4)	S1—C11—H111	109.5
C1—B6—B11	107.6 (4)	S1—C11—H112	109.5
B10—B6—B11	60.7 (3)	H111—C11—H112	109.5
B2—B6—B11	60.7 (3)	S1—C11—H113	109.5
F6—B6—B5	118.6 (4)	H111—C11—H113	109.5
C1—B6—B5	58.3 (3)	H112—C11—H113	109.5
B10—B6—B5	60.3 (3)	S1—C12—H121	109.5
B2—B6—B5	108.2 (4)	S1—C12—H122	109.5
B11—B6—B5	109.0 (4)	H121—C12—H122	109.5
B2—B7—B8	108.4 (4)	S1—C12—H123	109.5
B2—B7—B12	107.7 (4)	H121—C12—H123	109.5
B8—B7—B12	59.9 (3)	H122—C12—H123	109.5
B2—B7—B11	60.1 (3)	S1—C13—H131	109.5
B8—B7—B11	108.1 (4)	S1—C13—H132	109.5
B12—B7—B11	59.7 (3)	H131—C13—H132	109.5
B2—B7—B3	60.4 (3)	S1—C13—H133	109.5
B8—B7—B3	60.0 (3)	H131—C13—H133	109.5
B12—B7—B3	107.7 (4)	H132—C13—H133	109.5
